# Effect of cervical cerclage on uterine artery doppler parameters and small for gestational age births

**DOI:** 10.1038/s41598-025-19706-z

**Published:** 2025-10-05

**Authors:** Zeynep Kayaoglu Yildirim, Isil Turan Bakirci, Guray Tuna, Nihal Callioglu

**Affiliations:** 1 Division of Perinatology, Department of Obstetrics and Gynecology, Basaksehir Cam and Sakura City Hospital, Istanbul, Turkey; 2 Division of Perinatology, Department of Obstetrics and Gynecology, Gaziosmanpasa Training and Research Hospital, Istanbul, Turkey

**Keywords:** Cervical cerclage, Uterine artery doppler, Fetal development, Prophylactic cerclage, Ultrasound-based cerclage, Rescue cerclage, Maternal-fetal circulation, Outcomes research, Health care

## Abstract

To evaluate the effect of vaginal cervical cerclage on uterine artery Doppler parameters and its association with small for gestational age births across different clinical indications. This prospective observational study included 140 singleton pregnancies undergoing cervical cerclage, stratified by indication: prophylactic (*n* = 39), ultrasound-based (*n* = 62), and rescue cerclage (*n* = 39). Uterine artery pulsatility index was measured preoperatively, postoperatively, and at six weeks post-procedure. Fetal growth parameters were monitored throughout pregnancy, with births below the 10th percentile classified as small for gestational age. All procedures utilized McDonald’s technique at a single tertiary center. Postoperative pulsatility index increased significantly in the prophylactic cerclage group (*p* < 0.001), but showed minimal change in the ultrasound-based and rescue groups. PI values normalized in all groups by six weeks. Small for gestational age births occurred in 7.9% overall (prophylactic 12.8%, ultrasound-based 4.8%, rescue 7.7%), with no significant difference (*p* = 0.410). Postoperative pulsatility index values above the 95th percentile were significantly more common in the prophylactic group (30.8%) than in ultrasound-based (6.5%) or rescue cerclage groups (2.6%) (*p* < 0.001). Cervical cerclage may cause early, transient uterine hemodynamic changes, particularly in women undergoing prophylactic cerclage for a history of painless mid-trimester loss. These changes typically resolve within six weeks and are not associated with adverse fetal growth outcomes, supporting an adaptive rather than pathological response. In clinical practice, transient postoperative Doppler elevations should be interpreted in light of indication and timing rather than regarded as alarming. These findings provide reassurance for clinical counseling on cerclage safety and emphasize the importance of indication-specific Doppler monitoring to guide follow-up and patient management.

## Introduction

Preterm labor is one of the leading causes of perinatal mortality and long-term morbidity^1^. Cervical insufficiency, characterized by painless cervical dilatation, is a preventable cause of second-trimester pregnancy loss and spontaneous preterm birth^2^. Cerclage is an effective surgical method to prevent preterm labor in cases of cervical insufficiency^3^. Cerclage may be indicated based on the history, ultrasound findings, and cervical changes detected by physical examination. History-based cerclage is performed in women with a history of preterm delivery or second-trimester pregnancy loss due to cervical insufficiency, and ultrasound-based cerclage is performed in the presence of a short cervix (< 25 mm). In emergencies, such as acute cervical dilatation and bulging membranes, rescue cerclage is preferred. Cerclage may be vaginal or abdominal, with the abdominal approach reserved for failed vaginal cerclage or severe cervical shortening. Cerclage can be performed prophylactically or emergently. While prophylactic cerclage is usually performed at 12–14 weeks of gestation, emergency cerclage can be performed at up to 24 weeks of gestation in cases of cervical changes. Cerclage timing is an important factor that can affect maternal and fetal outcomes^4–6^.

Uterine artery Doppler ultrasound is an important method for the evaluation of maternal-fetal circulation. The pulsatility index (PI), which is used to evaluate uterine arterial blood flow, is an important parameter that indicates the vascular resistance of the uterine artery and changes in blood flow. The pulsatility index was calculated as the ratio of the difference between the systolic and diastolic blood flow velocities to the mean blood flow velocity. High PI values reflect increased vascular resistance and low PI values reflect decreased vascular resistance. During normal pregnancy, uterine artery PI values gradually decrease as the gestational age progresses. PI values, which are high in the first trimester (mean 1.5–2.5), show a significant decrease in the second trimester (mean 0.7–1.5) with the physiological transformation of the spiral arteries. This physiological decrease reflects increased uterine blood flow and improved maternal-fetal circulation^7–11^.

Normal uterine artery PI values during pregnancy are an important indicator of adequate uterine perfusion and optimal fetal development. Suture placement and mechanical manipulation of the cervix during cerclage application may affect uterine blood flow. Nemescu et al. evaluated the changes in uterine arterial blood flow with three-dimensional power Doppler in a case of modified laparoscopic transabdominal cerclage. In their study, they observed a decrease in the uterine artery PI values during the early post-cerclage period. They also reported that collateral circulation developed from the 16th gestational week and PI values were significantly lower in the uterine artery on the side where the placenta was located^12^. These findings reveal the hemodynamic effects of cerclage on maternal-fetal circulation.

No comprehensive study has compared the effects of different cerclage indications on uterine artery blood flow and birthweight outcomes. To fill this gap, we aimed to evaluate the effects of a vaginal cerclage procedure performed with different indications on maternal-placental-fetal circulation using the uterine artery mean PI and birthweight below the 10th percentile. We also examined the effects of preoperative and postoperative cervical length, cerclage week, and indications for uterine artery PI values.

Our hypothesis is that mechanical manipulation of the cervix caused by cerclage application may lead to a transient increase in uterine artery PI values in the early period, but this change may differ between indication groups and may return to normal over time. In addition, we anticipate that these vascular changes may have some effect on fetal growth parameters.

## Methods

This prospective observational study included 140 singleton pregnancies that underwent cervical cerclage at Cam and Sakura City Hospital. The study was approved by the Ethics Committee of Cam and Sakura City Hospital (KAEK/2022.06.180), and written informed consent was obtained from all participants in accordance with the Declaration of Helsinki.

Patients were stratified into three groups based on the clinical indication for cerclage:

Prophylactic cerclage (Group 1, *n* = 39): A history-indicated cerclage was performed between 12 and 14 weeks of gestation in women with previous painless second-trimester losses due to cervical insufficiency, in the absence of current cervical changes.

Ultrasound-based cerclage (Group 2, *n* = 62): An ultrasound-based cerclage was performed before 24 weeks for cervical length < 25 mm in women with a history of preterm birth before 34 weeks, or < 20 mm in those without such history.

Rescue cerclage (Group 3, *n* = 39): Emergency cerclage performed in cases of premature cervical dilatation with prolapse of fetal membranes into the vagina, typically performed up to 24 weeks of gestation.

Inclusion criteria comprised singleton pregnancies between 18 and 45 years of age with documented indication for cervical cerclage and provision of informed consent.

Exclusion criteria included chronic maternal diseases such as hypertension, cardiac disease, or diabetes mellitus; fetal aneuploidy or major structural malformations; prior cervical surgery; multiple pregnancies; smoking; cervicitis; and history of preeclampsia, intrauterine growth restriction, or small for gestational age delivery in previous pregnancies.

All cerclages were performed by one maternal-fetal medicine specialist using the McDonald technique under regional or general anesthesia. Following placement of ring forceps on the anterior and posterior cervical lips, cervical length and dilatation status were assessed. A suture was placed using either Prolene No. 1 or Mersilene tape, positioned as posteriorly as possible starting from the 12 or 1 o’clock position while carefully avoiding vascular structures at the 3 and 9 o’clock positions. After appropriate suture tightening and confirmation of cervical closure, hemostasis and suture integrity were verified.

Perioperative management included a single preoperative intravenous dose of cefazolin (1 g) and indomethacin, followed by daily vaginal progesterone (200 mg); bed rest was recommended for 24 h before resumption of light activity.

Comprehensive demographic and clinical data were systematically recorded, including maternal age, gravidity, parity, body mass index, preoperative and postoperative cervical length measurements, gestational age at cerclage, and birth outcomes including gestational age at delivery and birth weight. Small for Gestational Age (SGA) was defined as a birth weight below the 10th percentile for gestational age, while Intrauterine Growth Restriction (IUGR) was defined as pathologic fetal growth restriction with an estimated fetal weight (EFW) or abdominal circumference (AC) below the 10th percentile, accompanied by abnormal Doppler findings or other evidence of fetal compromise. In this study, all SGA cases were classified based solely on this weight criterion regardless of IUGR status, allowing us to evaluate the association between uterine artery Doppler changes and SGA. Definitions were based on ACOG Practice Bulletin No. 227 (2021)^13^ and ISUOG Practice Guidelines (2020)^14^.

Uterine artery Doppler evaluations were performed preoperatively, immediately postoperatively, and at 6 weeks by a single maternal-fetal medicine physician using standardized technique and identical equipment (Hitachi Aloka Arietta 70).

A transabdominal 3.5–5 MHz probe was used with the patient supine and bladder moderately filled. Uterine arteries were assessed distal to the internal cervical os near the iliac junction. Doppler angle was kept < 60°, and ≥ 3 consecutive high-quality waveforms were obtained for each artery. Mean PI was calculated from bilateral measurements to minimize variability.

At our institution, first-trimester preeclampsia screening routinely involves uterine artery Doppler assessment; aspirin prophylaxis (100 mg/day) is initiated when the uterine artery pulsatility index (PI) exceeds the 95th percentile. In this study cohort, no patients met this criterion, and none received aspirin.

Data were analysed using R version 4.3.1 (R Foundation for Statistical Computing, Vienna, Austria). Descriptive statistics are presented as mean ± standard deviation (SD) for normally distributed continuous variables and as median (interquartile range) for non-normally distributed variables. Categorical variables are summarised as counts and percentages. Normality was evaluated with the Shapiro–Wilk test and by visual inspection of Q–Q plots. When the normality assumption was violated, non-parametric tests were applied: the Wilcoxon signed-rank test for paired comparisons (e.g. pre- and postoperative uterine-artery pulsatility index [PI]) and the Mann–Whitney U test for independent samples. Differences in PI between (i) pre- and postoperative measurements and (ii) postoperative and 6-week follow-up measurements were assessed within each indication group. Categorical data were compared with the χ² test or Fisher’s exact test, as appropriate. Statistical significance was defined as *P* < 0.05.

A post-hoc power analysis was performed to evaluate the ability of the study to detect the observed change in uterine-artery PI before and after cerclage placement. Although the paired comparison employed the Wilcoxon signed-rank test because the data were non-normal, power was conservatively approximated with a paired-samples *t*-test model. With a sample size of 140, an α level of 0.05, and an observed mean difference of 0.08 (SD ≈ 0.48), the study had > 90% power to detect this difference. Calculations were performed using G*Power version 3.1.9.7.

## Results

A total of 140 singleton pregnancies met the inclusion criteria and were included in the final analysis. The baseline demographic and clinical characteristics are summarized in Table 1. The median maternal age was 29.0 years (interquartile range: 25–33), and over half of participants (53.6%) were nulliparous. Gravidity distribution showed 28.6% gravida 1, 25.0% gravida 2, and 46.4% gravida ≥ 3. The median BMI was 28.0 kg/m² (IQR: 24.8–31.2).

**Table 1:** Demographic and clinical characteristics of the study population.

Maternal age (years)	29.0 (25–33)
Gravidity	
1	40 (28.57%)
2	35 (25%)
≥ 3	65 (46.43%)
Parity	
0	75 (53.57%)
1	31 (22.14%)
≥ 2	34 (24.29%)
BMI	28.0 (24.8-31.24)
Cervical Length (mm)	12.0(2.0–25.0)
Gestational age at cerclage (weeks)	20.43(14.93–22.43)
Indication for cerclage	
Prophylactic	39 (27.86%)
Ultrasound based	62 (44.29%)
Rescue	39 (27.86)
Suture material	
Mersilene	57 (40.71%)
Prolene	83 (59.29%)
Postoperative cervical length (mm)	28.0 (22.0–33.0)
Preoperative uterine PI	1,02 (0.45–2.36)
Postoperative uterine PI	1,03 (0.60-2,68)
Uterine PI at 6 weeks	0,81 (0.52–3.95)
Gestational age at delivery (week)	36,29 (32.93-38.0)
Birth weight (g)	2775 (1885–3220)
Birthweight < 10th percentile	

Data are presented as median (Q1-Q3) for continuous variables and proportions (%) for categorical variables.

BMI: Body Mass Index

PI: Pulsatility Index

Q1-Q3: First and third quarter

The cerclage procedure was performed at a median gestational age of 20.4 weeks (IQR: 14.9–22.4), varying significantly by indication: prophylactic cerclages were placed earlier (median 14.2 weeks) and rescue cerclages later (median 22.8 weeks).Median cervical length increased from 12.0 mm (IQR: 2.0–25.0) preoperatively to 28.0 mm (IQR: 22.0–33.0) postoperatively (*p* < 0.001).

Regarding pregnancy outcomes, the median gestational age at delivery was 36.3 weeks (IQR: 32.9–38.0), and the median birth weight was 2775 g (IQR: 1885–3220). The distribution of cerclage indications was relatively balanced, with 39 patients (27.86%) in the prophylactic group, 62 patients (44.29%) in the ultrasound-based group, and 39 patients (27.86%) in the rescue group.

Pre- and postoperative uterine artery PI values by indication are shown in Table 2. A significant difference in PI changes was observed between the indication groups (*p* < 0.001). The most pronounced increase in PI was noted in Group 1 (prophylactic cerclage) with a median change of -0.26 (IQR: 0.31), while only minor changes were seen in Group 2 (ultrasound-based cerclage) and Group 3 (rescue cerclage) with median changes of -0.02 (IQR: 0.18) and − 0.01 (IQR: 0.15), respectively.

**Table 2:** Mean difference in preoperative and postoperative uterine PI by ındication, gestational age at cerclage, cervical length, and suture material.

Variables	Mean ± SD	*p*-value
Indication for cerclage		< 0.001
Prophylactic	– 0.26 (0.31)	
Ultrasound based	– 0.02 (0.18)	
Rescue	– 0.01 (0.15)	
Gestational age at cerclage	0.288	< 0.001
Cervical length	– 0.409	< 0.001
Suture material		0.165
Mersilene	– 0.08 (– 0.33)	
Prolene	– 0.01 (– 0.22)	

Data are presented as Mean ± SD. Differences between preoperative and postoperative uterine PI were analyzed using appropriate statistical tests.

PI: Pulsatility Index

SD: Standard Deviation

Overall PI values (Table 3) increased significantly from 1.12 (IQR: 0.43) preoperatively to 1.20 (IQR: 0.54) postoperatively (*p* < 0.001), then decreased to 0.81 (IQR: 0.52–3.95) by postoperative week 6 (*p* < 0.001).

**Table 3:** Comparison of preoperative and postoperative uterine Pi across the study population.

	Mean	SD	*p*-value
Preoperative PI	1.12	0.43	**< 0.001**
Postoperative PI	1.20	0.54	

Preoperative PI	Postoperative PI	*p*-value
1.12 ± 0.43	1.20 ± 0.54	**< 0.001**

Data are presented as mean (SD). Differences between preoperative and postoperative uterine PI were analyzed using appropriate statistical tests.

PI: Pulsatility Index

SD: Standard Deviation

The rates of uterine artery PI above the 95th percentile in the postoperative period are shown in Table 4. The highest rate was observed in Group 1 (30.8%, *n* = 12), followed by Group 2 (6.5%, *n* = 4), and Group 3 (2.6%, *n* = 1). The differences between the groups were statistically significant (*p* < 0.001). Analysis of factors associated with high PI values revealed that patients with PI > 95th percentile had significantly earlier gestational age at cerclage (median 16.0 vs. 19.9 weeks, *p* = 0.002) and shorter preoperative cervical length (median 8.0 vs. 12.0 mm, *p* = 0.005). Suture material type did not influence high PI occurrence (Mersilene 17.5% vs. Prolene 8.4%, *p* = 0.092).

**Table 4:** Comparison of postoperative uterine artery pi > 95th percentile by indication, gestational age at cerclage, cervical length, and suture material.

	PI ≤ 95th Percentile (*n*, %)	PI > 95th Percentile (*n*, %)	*p*-value
Indication for cerclage			**< 0.001**
Prophylactic	27 (69.2%)	12 (30.8%)	
Ultrasound based	57 (93.4%)	4 (6.6%)	
Rescue	38 (97.4%)	1 (2.6%)	
Gestational age at cerclage (weeks, Mean ± SD)	19.86 (3.93)	16.01 (4.10)	**0.002**
Cervical length (mm, Median (Q1-Q3))	10.0 (0.0–46.0)	32.0 (0.0–44.0)	**0.005**
Suture material			0.092
Mersilene	47 (82.5%)	10 (17.5%)	
Prolene	75 (91.5%)	7 (8.5%)	

Continuous data are presented as Mean ± SD and median (Q1-Q3). Categorical variables are presented as frequencies and row percentages. Differences between preoperative and postoperative uterine PI were analyzed using appropriate statistical tests.

PI: Pulsatility Index

SD: Standard Deviation

Q1-Q3: First and third quarter

The incidence of small for gestational age births was 7.86% (*n* = 11) across the entire study population, which is comparable to expected population rates. The distribution analysis by cerclage indication is presented in Table 5, which revealed a numerically higher rate in the prophylactic group (12.8%, *n* = 5) compared to the ultrasound-based group (4.8%, *n* = 3) and rescue group (7.7%, *n* = 3), though this difference did not reach statistical significance (*p* = 0.410). Additional analysis showed no significant differences in gestational age at cerclage (*p* = 0.605) or preoperative cervical length (*p* = 0.610) between SGA and normal birth weight groups. Suture material type did not influence SGA occurrence (*p* = 0.355).

**Table 5:** Comparison of birthweight < 10th percentile by indication, gestational age at cerclage, cervical length, and suture material.

	Birthweight ≥ 10th Percentile (*n*, %)	Birthweight < 10th Percentile (*n*, %)	*p*-value
Indication for cerclage			0.410
Prophylactic	34 (87.2%)	5 (12.8%)	
Ultrasound based	59 (95.2%)	3 (4.8%)	
Rescue	36 (92.3%)	3 (7.7%)	
Gestational age at cerclage (weeks, Median (Q1-Q3)	20,4 (15,4–22,4)	20,4 (13,4–22,4)	0.605
Cervical length (mm, Median (Q1-Q3)	27,4 (8,7)	25,5 (8,5)	0.610
Suture material			0.355
Mersilene	51 (89.5%)	6 (10.5%)	
Prolene	78 (94.0%)	5 (6.0%)	

Continuous data are presented as Median (Q1-Q3). Categorical variables are presented as frequencies and row percentages. Differences in fetal growth outcomes were analyzed using appropriate statistical tests.

PI: Pulsatility Index

Q1-Q3: First and third quarter

## Discussion

In this study, we evaluated the effects of vaginal cerclage applied for different indications on uterine artery blood flow and fetal development. Our findings showed that cerclage affected uterine artery PI differently across indication groups: a significant postoperative increase was observed in the prophylactic group, minimal changes were noted in the ultrasound-based and rescue groups, and PI values normalized in all groups by week 6.

The significant increase in PI observed in the prophylactic cerclage group can be explained by various factors. Prophylactic cerclage is usually performed at early gestational weeks, and the cervix retains its normal anatomical structure. In recent years, there have been various discussions on the effects of cervical cerclage on uterine artery Doppler parameters and fetal growth. In a letter to the editor questioning the pressure of laparoscopic cerclage on the uterine artery and its potential effects on fetal growth, Su et al. suggested that this procedure might adversely affect fetal development^15^. However, this view has not been confirmed in larger-scale randomized trials, and it is not yet known whether it has an effect similar that to of transvaginal cerclage. Therefore, studies evaluating the effects of cerclage on uterine artery hemodynamics need to be compared with respect to different surgical techniques.

The mechanical effect of surgical manipulation may be more pronounced because of intact tissue integrity. Reorganization of the cervical vascular structures is not yet complete in the early gestational weeks, and compensatory mechanisms have not fully developed in the cervical tissue, which was encountered with surgical intervention for the first time. In this period, when there is no cervical dilatation, tissue resistance is higher and more mechanical force is required to tighten the suture, which may cause a greater compression effect on the cervical vascular structures.

The lack of significant PI changes in the ultrasound-based and rescue cerclage groups may be related to the different anatomical and physiological conditions in these groups. Especially in the rescue cerclage group, the anatomy of the cervix was altered due to cervical dilatation and prolapse of the fetal membranes. Collateral circulation due to these changes and less resistance of the tissue to surgical manipulation may limit its effect on the vascular system.

The decrease in PI values observed in all groups at postoperative week 6 indicated vascular adaptation to surgical trauma. Our findings support the vascular adaptation process reported by Nemescu et al. in patients undergoing modified laparoscopic cerclage. They reported that collateral circulation developed from the 16th gestational week, and the PI values returned to normal over time^12^. This is consistent with the normalization of PI values at postoperative week 6 observed in our study. These findings support the hypothesis that transient hemodynamic changes that occur after cerclage can return to normal within weeks following pregnancy.

Although the rate of fetal SGA was higher in the prophylactic cerclage group (12.8%) compared to the ultrasound-based group (4.8%) and rescue group (7.7%), no statistically significant difference was found (*p* = 0.410). This suggests that prophylactic cerclage at early gestational weeks and the associated PI changes do not have a significant negative effect on fetal growth. This finding aligns with a recent study by Brooks et al., which investigated whether short cervix patients undergoing ultrasound-indicated cerclage had an increased risk of delivering a SGA newborn^16^. Their findings suggest that while cerclage may influence uterine artery hemodynamics, the long-term impact on fetal growth remains uncertain. However, long-term follow-up studies in larger patient series are required to obtain more definitive results.

This numerical difference in SGA rates warrants further consideration of underlying causes.

Importantly, the numerically higher SGA rate in the prophylactic group may reflect pre-existing placental pathophysiology rather than the cerclage procedure itself. Patients requiring prophylactic cerclage often have a history of recurrent pregnancy loss or extremely preterm birth—conditions associated with subclinical placental insufficiency, impaired spiral artery remodeling, and maternal vascular pathology^17–19^. This background vulnerability, combined with the early timing of cerclage during critical windows of placental development, could potentially contribute to later fetal growth restriction.

Our findings offer practical guidance for clinical practice and patient counseling. Healthcare providers can reassure patients that, while cerclage procedures may cause temporary alterations in uterine blood flow—particularly in prophylactic cases—these changes are expected, self-limited, and do not appear to compromise fetal well-being. The transient PI elevations observed, especially in the prophylactic group, may reflect a vascular adaptation to surgical manipulation rather than pathological placental insufficiency. Importantly, these findings emphasize the need to avoid unnecessary interventions (such as unwarranted aspirin use) or patient anxiety based solely on transient abnormalities, particularly in those undergoing prophylactic cerclage. The six-week normalization timeframe observed in our study provides a practical benchmark for clinical follow-up protocols. The differential hemodynamic responses between indication groups suggest that patients undergoing prophylactic cerclage may benefit from closer surveillance during the immediate postoperative period, although long-term intensive monitoring appears unwarranted in light of the consistent recovery pattern.

## Limitations

This study has several limitations. First, its single-center design and relatively small sample size limit generalizability. Postoperative uterine artery PI values were followed for only 6 weeks, which restricts the evaluation of long-term vascular changes. Extended follow-up of Doppler parameters into the third trimester would allow for long-term monitoring of complications and fetal outcomes, offering a more comprehensive understanding of maternal-fetal circulatory changes after cerclage.

Another limitation is the absence of a control group of high-risk women who met the criteria for cerclage but were managed conservatively without surgery. Including such a group could have strengthened causal inferences. However, this was ethically and clinically challenging given current guideline recommendations. As a result, the observed Doppler changes and fetal growth outcomes should be interpreted with caution.

The limited number of SGA cases also prevented multivariable adjustment for potential confounders, emphasizing the need for larger cohorts.

Finally, additional demographic characteristics such as ethnicity, socioeconomic status, and education level could not be analyzed due to the retrospective nature of data collection. This limitation was recognized and considered in the interpretation of our findings.

## Conclusion

In this study, the effect of vaginal cerclage on uterine artery blood flow varied by indication. The early increase in PI observed, particularly in the prophylactic cerclage group, reflects a cervical vascular response to surgical manipulation. Our findings align with early vascular changes reported in previous studies; however, these changes were transient and normalized by postoperative week 6. These findings support the continued use of cerclage for appropriate clinical indications while providing evidence-based reassurance regarding hemodynamic safety concerns. Furthermore, they underscore the importance of indication-specific hemodynamic monitoring to guide postoperative follow-up and patient management.

Although fetal growth restriction was more frequent in the prophylactic group, the difference was not statistically significant, suggesting no adverse effect on fetal growth.

Overall, this study provides important data for evaluating the hemodynamic effects of vaginal cerclage and its relationship with fetal development, with confirmation through prospective, multicenter, long-term studies are needed to achieve a more comprehensive understanding of the effects of cerclage on maternal-fetal circulation.

## Data Availability

The data that support the findings of this study are available from the corresponding author upon reasonable request. Access to the data will be considered following review of a detailed research proposal to ensure appropriate use and protection of patient information.
